# Fiducial Objects: Custom Design and Evaluation

**DOI:** 10.3390/s23249649

**Published:** 2023-12-06

**Authors:** Pablo García-Ruiz, Francisco J. Romero-Ramirez, Rafael Muñoz-Salinas, Manuel J. Marín-Jiménez, Rafael Medina-Carnicer

**Affiliations:** 1 Departamento de Informática y Análisis Numérico, Edificio Einstein, Campus de Rabanales, Universidad de Coŕdoba, 14071 Córdoba, Spain; pgruiz@uco.es (P.G.-R.); fj.romero@uco.es (F.J.R.-R.); rmedina@uco.es (R.M.-C.); 2 Instituto Maimónides de Investigación en Biomedicina (IMIBIC), Avenida Menéndez Pidal s/n, 14004 Córdoba, Spain

**Keywords:** fiducial object, camera pose estimation, fiducial marker

## Abstract

Camera pose estimation is vital in fields like robotics, medical imaging, and augmented reality. Fiducial markers, specifically ArUco and Apriltag, are preferred for their efficiency. However, their accuracy and viewing angle are limited when used as single markers. Custom fiducial objects have been developed to address these limitations by attaching markers to 3D objects, enhancing visibility from multiple viewpoints and improving precision. Existing methods mainly use square markers on non-square object faces, leading to inefficient space use. This paper introduces a novel approach for creating fiducial objects with custom-shaped markers that optimize face coverage, enhancing space utilization and marker detectability at greater distances. Furthermore, we present a technique for the precise configuration estimation of these objects using multiviewpoint images. We provide the research community with our code, tutorials, and an application to facilitate the building and calibration of these objects. Our empirical analysis assesses the effectiveness of various fiducial objects for pose estimation across different conditions, such as noise levels, blur, and scale variations. The results suggest that our customized markers significantly outperform traditional square markers, marking a positive advancement in fiducial marker-based pose estimation methods.

## 1. Introduction

Camera pose estimation is the task of determining a camera’s spatial position and orientation in relation to a known reference system from an image. The significance of this task reaches into multiple domains, including robotics, medical imaging, and augmented reality [1,2,3,4]. A common strategy to solve the task involves introducing a known object as a spatial reference into the scene.

In the evolving field of camera pose estimation, diverse methodologies have been explored, each with unique strengths and challenges. Feature-based approaches, such as the system detailed by Campos et al. [5], have been prominent due to their precision and adaptability in identifying and tracking distinctive image features like edges and corners. However, they often struggle in environments with repetitive patterns or sparse textural details. Advancements in artificial intelligence have led to innovative solutions to counter these limitations. For example, methods like the one proposed by Li et al. [6] utilize direct image analysis for camera pose estimation, but these also face difficulties in complex settings. This necessitates exploring alternative methodologies, such as fiducial markers, which offer a more reliable camera pose estimation method under such challenging visual circumstances.

Fiducial markers, such as ArUco and Apriltag [7,8] have gained popularity for their utility and performance, leading to their widespread use in camera pose estimation. A fiducial marker is a pattern (generally a square) of black-and-white elements that encodes information representing unique identifiers. Fiducial markers can be detected efficiently in images, using their four vertices to estimate the camera pose. However, calculating the pose from a single marker has limitations regarding the accuracy and the range of viewing angles.

To address these limitations, some authors [9,10,11,12] have proposed crafting three-dimensional objects to which squared fiducial markers are attached. We call them *fiducial objects.* The object can be used to estimate the camera position from a broader range of viewpoints than a single marker. Also, since multiple markers may be visible at the same time, the precision of the estimation can be improved. However, the proposed approaches have some limitations. First, it is required to know the markers’ relative position with high precision to achieve good accuracy, but many of the current proposals do a poor job by manually estimating them. Second, they all rely on squared markers. A couple of clear examples are Dodecapen [10] and the work proposed by Xiang et al. [12], whose authors designed polyhedrons with ArUco squared markers attached. Instead, it would have been more natural to use pentagonal markers to fit the dodecahedron’s faces properly. In that sense, Jurado et al. [13] proposed a method to create custom markers, avoiding the limitation of squared markers. By following a set of rules, one can create a set of markers with unique identifiers but with any desired style.

**Figure 1:**
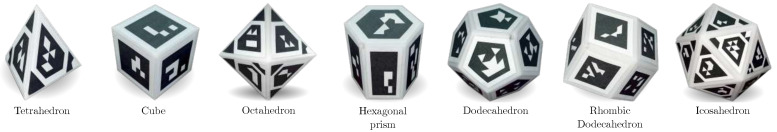
Fiducial objects created in the study.

This paper proposes a general method to create fiducial objects with custom markers that can be adapted to the needs of specific use cases. We expand the work of Jurado et al. to the 3D domain by proposing a method to create custom fiducial objects. Figure 1 shows some fiducial objects that can be created with our method.

The main contributions of this paper are four. First, we propose a general method to build fiducial objects with customized markers that properly fit their faces. Unlike traditional squared markers, our proposal allows for adjusting the object’s shape to capture all the available space better. Due to having more space, our markers can encode more bits and be detected at larger distances. Second, we provide a method to accurately estimate the object’s configuration (i.e., the positions of the markers) from images of the object taken from multiple viewpoints. Third, this paper presents an initial study examining various shapes to determine the optimal choice for creating a fiducial object for camera/object pose estimation. To do so, we evaluate the performance of multiple objects created with our method under different conditions, such as noise, blur, and scale changes. As the final contribution, our code and dataset are set public for other researchers to use in their work freely. We provide the tools and tutorials to easily design, create, and test your own fiducial objects, even for non-technical researchers.

The rest of the paper is structured as follows. Section 2 presents the works most related to ours. Section 3 explains the method proposed, while Section 4 explains the experiments carried out. Finally, Section 5 draws some conclusions.

## 2. Related Works

Squared fiducial markers [7,8,14,15] are the most popular markers used for camera pose estimation. This type of marker consists of a square border for contour detection and an inner region for marker identification. The presence of four prominent corners allows for the detection of the camera pose. Other shapes, such as circles, have also been proposed as markers, for instance, WhyCode [16] and RuneTag [17]. Unlike square-shaped markers, they offer a different way of encoding information and can be more suitable for specific applications. In a comparative study by Jurado et al. [18], the effectiveness of different types of fiducial markers is analyzed, observing that, in general, ArUco [7] is the best-performing fiducial marker system.

A main drawback of the previously referenced markers is that their design is fixed and, in many cases, too industrial. Other authors [13,19] have proposed customizable markers that can vary in shape and color to look like, for instance, a unique design or logo, making them useful for branding or advertising. They can be designed, following a set of rules, to be fashionable and thus more readily accepted in non-industrial environments. The first proposal of this approach was VuMark [19], a proprietary technology that may not be suitable for scientific research applications where open-source or non-proprietary solutions are preferred. The other alternative is Jumark [13], an open-sourced customized fiducial markers system proposing a method for generating markers with various shapes, enhancing the adaptability for commercial applications.

As the field of fiducial markers continues to evolve, deep learning has emerged as a promising technique to address some of the existing challenges. Leveraging neural networks, researchers are exploring ways to enhance the detection and tracking capabilities of camera systems, even in complex scenarios characterized by occlusions or variable lighting [20,21]. Despite its potential, applying deep learning in marker detection is not without its own challenges. Concerns have been raised regarding computational efficiency and the dependency on extensive training data. Such systems often demand significant computational resources, which may limit their practicality in environments with limited resources. Additionally, the robustness and reliability of AI-driven markers in diverse and unpredictable conditions continue to be areas needing further research and development.

Estimating the pose of an object from a single marker is suboptimal. First, it limits the range of viewpoints from which it can be detected. Second, pose estimation from planar surfaces suffers from the ambiguity problem [22], i.e., under certain circumstances, it is impossible to estimate the pose’s rotational component unambiguously. Therefore, it is preferable to use multiple markers in the object that needs to be tracked.

Several works have proposed different approaches for estimating the pose of an object using multiple planar markers. A system that combines ArUco fiducial markers with a fiducial object for pose estimation, employing an array of cameras, is introduced by Sarmadi et al. [11]. Their approach determines the extrinsic parameters of the cameras and the relative poses between the markers and cameras in each image. Additionally, their technique enables the automatic acquisition of a three-dimensional arrangement for any set of markers. The main drawbacks of their work are that it is limited to squared planar markers and is only evaluated on one type of object under ideal conditions.

In the realm of alternative strategies, a noteworthy method is introduced by Jiang et al. [9]. They propose a ball-shaped object designed to be detectable and trackable across a wide range of orientations by utilizing circular markers affixed to it. In addition, they propose a novel algorithm for detecting these circular markers that aims to enhancing the accuracy and robustness of the detection process. Nevertheless, their work presents several limitations, including the absence of comparative analyses with different structures, the reliance on a stereo vision system, and the absence of a detailed, replicable methodology to reproduce their results. These factors, in turn, pose challenges for the broader application and validation of their proposed method.

Another system incorporating multiplanar fiducial markers is proposed by Wu et al. [10]. They introduce DodecaPen, a strategy for calibrating and tracking a 3D dodecahedron adorned with ArUco markers on its surfaces. This system facilitates real-time camera pose estimation using a single camera, which is employed to create a digital wand. Furthermore, they devise a series of methods for estimating the 3D relative positions of the markers on the dodecahedron. To counteract motion blur, a corner tracking algorithm is utilized. However, they do not provide code or binaries to reproduce their results.

A comparable solution has been presented by Chen et al. [1], wherein they introduce a tracking block comprising nine ArUco markers with the primary objective of addressing occlusion issues during the tracking process of surgical instruments. Additionally, they employ multiple cameras simultaneously to enhance accuracy. Other authors have also proposed methods for creating polyhedra using ArUco markers on their faces [23,24]. Xiang et al. [12] proposed five different Platonic solids, each adorned with ArUco markers on their surfaces, similar to the DodecaPen. These polyhedrons are intended for use as pose estimation objects. Moreover, they propose various strategies to counteract illumination, occlusion, and jitter issues. Nonetheless, how they calibrate the object is missing in the paper, and a study of the performance under different stressing situations, like noise or blur, is needed.

Despite the advances made by these studies, their methodologies exhibit certain common drawbacks. Firstly, they do not efficiently utilize the available space of the object since they are all limited to the squared shape of the markers employed. For instance, Dodecapen employs square fiducial markers on pentagonal faces. We propose the creation of custom fiducial objects by extending the ideas proposed in JuMark to the three-dimensional space. Therefore, the markers employed in our fiducial objects can be better adapted to the shape of their faces. Secondly, in many previous works, estimating the relative position between the object’s markers is either manual, unknown [12], or based on a complex system unavailable in most labs [9].

In a preliminary work [25] of ours, we presented a method for generating a dodecahedron composed of pentagonal fiducial markers. This work extends our preliminary study in many ways. First, we allow the creation of arbitrary customizable fiducial objects. Second, we propose a method to estimate the marker position in the object by simply employing images of the object taken from multiple viewpoints. This can be seen as an extension of the work of Muñoz et al. [26] to markers of an arbitrary shape. Third, the absence of accessible source code or binaries can hinder other researchers from utilizing or evaluating a work. We set out code that is public and open-source for other researchers to benefit from it. Also, we provide tools and tutorials for non-technical researchers to create and use custom fiducial objects in their work easily.

## 3. Proposed Method

This section describes the methodology proposed to create, detect, and estimate the pose of fiducial objects. We propose the term *fiducial object* to indicate a three-dimensional object with multiple polygonal faces with attached fiducial markers (Figure 1). To adapt to the different possible polygonal faces of a fiducial object, we employ custom marker *templates*, as proposed by Jurado et al. [13], instead of using squared markers like ArUco or AprilTags. A template allows us to create multiple markers with similar appearances but different IDs so that each one of them can be later detected in images uniquely.

Estimating the relative pose of a fiducial object to a camera from an image of it is a problem known as the Perspective-n-Problem [27], which is solved using a set of 3D-2D correspondences between the object’s points and their corresponding projections in the image.

Although designed by computer and 3D printed, the markers of objects are printed on paper and manually glued to their faces. Consequently, the actual three-dimensional position of the markers can not be precisely known. We propose a method to obtain a precise estimation of the markers using a set of images from different viewpoints through a global optimization process.

This section is structured as follows. First, Section 3.1 explains the basis of custom marker templates, and Section 3.2 explains how they are detected in images. Then, Section 3.3 describes the mathematical formulation of a fiducial object, and, finally, Section 3.4 explains how to obtain the actual fiducial object configuration from images of it.

### 3.1. Design of Custom Markers

**Figure 2:**
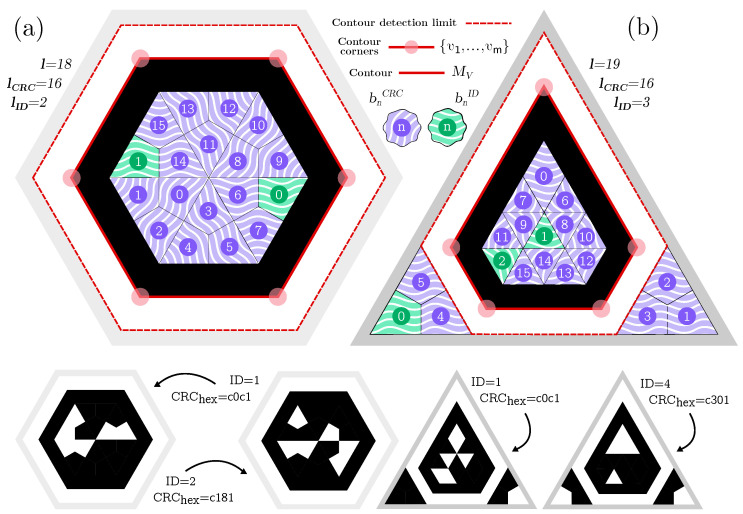
Detailed marker schemes: (**a**) prism’s hexagon marker scheme; (**b**) tetrahedron’s triangular marker scheme (See text for further details).

This subsection describes the methods used to design and detect custom markers in the polygonal faces of fiducial objects. Our approach is based on the work proposed by Jurado et al. [13], who proposes a method to design markers of arbitrary convex polygons. By providing a template that defines the marker boundary and the regions containing the bits, it is possible to automatically generate a set of unique markers with similar appearances, as shown in Figure 2.

A marker template can be denoted as the following tuple:

T={W,B},

where *W* is the polygon enclosing the marker such that

(1) $$W=\{\left\{w_{i}\right\},i\in \left[1,∆,n\right],\forall n\geq 4,w_{i}=\left(x_{i},y_{i}\right)\in R^{2}\},$$

where $w_{i}$ denotes the polygon vertices relative to the center of the marker. At least four vertices are required to estimate its three-dimensional position with respect to the camera.

The template polygon *W* has an internal black border surrounded by a white border to allow its reliable detection, as will be explained later. The rest of the space in the polygon is employed to encode information (bits) to identify each marker uniquely and for error detection. The template bits are denoted as follows:

(2) $$B=\{\left\{b_{j}\right\},j\in \left[1,∆,i,∆,l\right],b_{j}\in \left\{0,1\right\}\},$$

where $b_{j}|1\leq j\leq i$ are the bits used for identification since each marker has a unique ID. Then, $b_{j}|i<j\leq l$ represents the bits used for cyclic redundancy checking (CRC). Having as many CRC bits as possible is preferable to minimize the likelihood of false positives. However, this reduces the number *i* of identification bits and consequently the number of unique markers. In general, the total number of different markers is $2^{i}$. Each possible state of the marker bits is represented by a color chosen in the marker template at the design time. While black and white colors have been employed in Figure 2, they can be replaced by other colors with enough contrast.

### 3.2. Detection of Custom Markers

Marker detection in an image follows the philosophy proposed in the work of Jurado et al. [13]. Given an input image, the marker detection process is described as follows:

1. *Image segmentation*. A local adaptive thresholding filter is used to extract the image contours. The mean brightness of each pixel’s neighbor is compared to a threshold value to decide whether the pixel belongs to a border. This method has proven robust to irregular lighting conditions [7].
2. *Contour extraction and refinement*. Contours are extracted using the Suzuki and Abe method [28], and then a polygonal approximation [29] is applied to them. Only polygons *Q* with the same number of corners as our template polygon *W* are possible candidates to be valid markers; thus, the rest are discarded.
3. *Corner refinement.* For each marker candidate, we perform a refinement to find its corners accurately. This process improves the precision of the pose estimated and the chances of correct marker identification in the next step. The method employed first consists of calculating the lines’ equations that define the markers’ segments from the contour points. Then, we calculate their intersections and employ them as the new marker corners instead of the results from the polygonal approximation.
4. *Marker identification*. We run a set of tests for each candidate polygon *Q* to determine whether it is a valid marker generated with our template T. We compute the homography that maps the marker template vertices to the rotated vertices for every possible clockwise rotation of its vertices. The homography is employed to estimate the center of each bit in the image for that particular rotation to obtain its colors. We know the colors of a valid marker form a bimodal distribution; thus, we calculate the mean and use it to decide which are zeros or ones. If the sequence of bits belongs to a valid one from our template, then the marker is considered valid. In other words, if the identification bits produce the correct CRC, it is a valid marker.

This process results in the set of markers in an image, including the coordinates of their vertices.

Finally, let us remark that it is possible that one wants to define a fiducial object using markers of different templates (i.e., the hexagonal prism of Figure 1). In that case, the above algorithm can be easily modified to detect markers from multiple templates in an image.

### 3.3. Fiducial Objects

We represent a fiducial object

(3) $$O=\{\left\{m_{i}\right\},m_{i}=\left(v_{i}^{1},\ldots ,v_{i}^{n}\right)\}$$

as a set of custom markers $m_{i}$, arranged in a known position with respect to a common reference system, where $v_{i}^{j}\in R^{3}$ are the vertices of the marker. Let us assume, for now, that the vertices are precisely known. Later, we will explain how they can be obtained from images of the actual fiducial object.

The relative pose ϕ of the object with respect to the camera can be obtained from the vertices of its markers and its 2D observations in the image using the perspective-n-point (PnP) method, which consists of minimizing the reprojection error. Let us denote

(4) Ψ(δ,ϕ,p),

the function that obtains the two-dimentional projection in $R^{2}$ of a three-dimensional point $p\in R^{3}$ in a camera with intrinsics params δ after applying the rigid transform ϕ∈SE(3) to the point *p*.

Using the method described in Section 3.2, it is possible to detect the visible markers of the object in an image, thus obtaining a set of 3D-2D correspondences, i.e., $v_{i}^{j}\to \Psi \left(\delta ,\phi ,v_{i}^{j}\right),\forall j,i$.

If we denote by

(5) $$M^{f}=\left\{\left\{m_{i}^{f}\right\},m_{i}^{f}=\left(u_{i}^{1},\ldots ,u_{i}^{n}\right)\right\}$$

the marker set detected in the image *f*, where $u_{i}^{j}$ represents its 2D vertices, we can estimate the error of a given pose ϕ as the reprojection error

(6) $$E\left(\phi \right)=\sum_{m_{i}^{f}\in M^{f}}\sum_{j=1}^{n}\Psi \left(\delta ,\phi ,v_{i}^{j}\right)-u_{i}^{j}.$$

Then, finding the best pose $\hat{\phi }$ consists of minimizing the reprojection error so that

(7) $$\hat{\phi }=\underset{\phi }{arg min}E\left(\phi \right).$$

The markers detected are employed to obtain an initial estimation of the object pose using Equation (6), but the markers on faces that are somewhat perpendicular to the camera plane may not be identified because their bits are concentrated in a very small image area. However, the contour of these markers is sometimes detected and can be used to refine the pose even further. Since the initial pose allows us to calculate where all the markers should be in the image, we can spot the marker candidates corresponding to the object’s markers that the detection algorithm has not initially identified and use them to obtain a refined object pose.

### 3.4. Precise Estimation of the Fiducial Object Configuration

**Figure 3:**
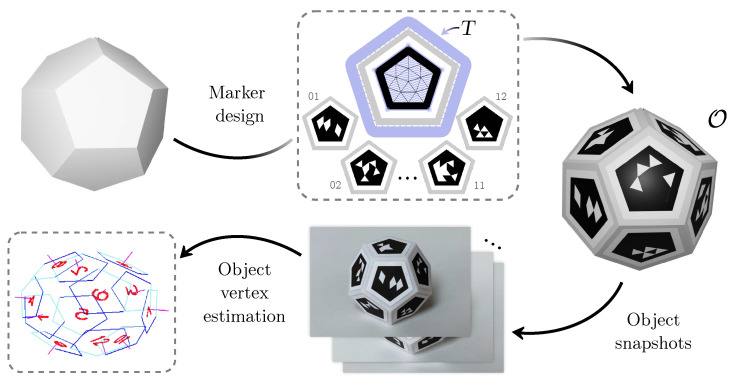
Simplified pipeline for proposed object creation (See text for further details).

Although objects can be designed with a computer and 3D printed, the markers are probably manually placed on the surfaces of the fiducial object; thus, the three-dimensional position of the vertices is not known with precision, and the pose estimated is subject to inaccuracies. To solve that problem, we explain how to obtain the coordinates of the object’s vertices with high precision using a set of images. The complete process is visually summarized in Figure 3.

Let us denote F={f} to a set of images of the object where at least two markers are detected in each image. We shall then estimate the relative pose $\phi_{i}^{f}$ of each marker with respect to the camera and

(8) $$\Phi^{f}=\left\{\phi_{i}^{f}\right\},$$

to the poses of the markers $m_{i}^{f}$ detected in *f* with respect to to the camera reference system. Please note that each marker has a different orientation, and thus its pose differs from the poses of the rest of the markers.

We aim to estimate the pose $\phi_{i}$ of each marker $m_{i}$ with respect to a common reference system to all markers, i.e., the object’s reference system. To do so, we first calculate the pairwise relationships between the markers in all images creating a pose quiver where nodes represent markers and vertices relative poses $\phi_{i,j}$ between them. The pose $\phi_{i,j}$ represents the SE(3) transformation moving the vertices of marker $m_{i}$ from its own reference system to the reference system of the marker $m_{j}$. From image *f*, we obtain all possible pairwise combinations of transforms $\phi_{i,j}^{f}$ between the markers found. Then, we shall denote the quiver vertices between nodes *i* and *j*

(9) $$\Phi_{i,j}^{F}=\left\{\phi_{i,j}^{f}\right\},$$

as the set of pairwise transforms observed in all the images of F.

The quiver is then converted into a directed pose graph where the vertices are the mean transform ${\hat{\phi }}_{j,i}$ for each pair of nodes, and nodes represent markers. Then, the graph is employed to obtain an initial estimation of the pose ${\hat{\phi }}_{i}$ of each marker $m_{i}$ with respect to a common reference system, as follows. First, we pick one marker, $m_{0}$, as the global reference system. Then, we calculate the pose of the rest of the markers by obtaining the minimum spanning tree and concatenating their pairwise transforms. For instance, if $\left\{m_{0},m_{b},m_{c},\ldots ,m_{k},m_{j},m_{i}\right\}$ is the path of the minimum set of concatenations leading to the reference marker $m_{0}$, the initially estimated pose of the marker $m_{i}$ is obtained as

(10) $${\hat{\phi }}_{i}={\hat{\phi }}_{0,b}{\hat{\phi }}_{b,c}\ldots {\hat{\phi }}_{k,j}{\hat{\phi }}_{j,i}.$$

We shall define $\hat{\Phi }=\left\{{\hat{\phi }}_{i}\right\}$ as the set of initial estimations of marker poses of the graph.

These initial solutions are refined considering all observations in the images F, i.e., a global optimization. To do so, let us define

(11) $$V\left({\hat{\phi }}_{i}\right)=\left({\hat{v}}_{i}^{1},\ldots ,{\hat{v}}_{i}^{n}\right),$$

as three-dimensional coordinates of the vertices of a marker given its pose ${\hat{\phi }}_{i}$ with respect to the object reference system.

The global optimization goal then becomes the problem of estimating the final poses Φ by minimizing the reprojection errors of their vertices in all the images of F. Since the vertices are referred to as the common reference system of the object, we shall denote by $\phi^{f}$ the object’s pose with respect to the image *f*. Then,

(12) $$\Psi (\delta ,\phi^{f},{\hat{v}}_{i}^{j})$$

denotes the projection of the vertex ${\hat{v}}_{i}^{j}$ in the image *f*, assuming that the object’s common reference system is placed at the position $\phi^{f}$ with respect to the image.

Finally, we can define the global optimization problem as

(13) $$\Phi =\underset{\hat{\Phi },\Phi^{F}}{arg min}\sum_{f}\sum_{M^{f}}\sum_{j}\Psi \left(\delta ,\phi^{f},{\hat{v}}_{i}^{j}\right)-u_{i}^{j},$$

where $\Phi^{F}=\left\{\phi^{f}\right\}$ represents the set of object poses with respect to the images in F. Equation (13) can be efficiently optimized using a sparse graph optimization approach [30].

## 4. Experiments

**Figure 4:**
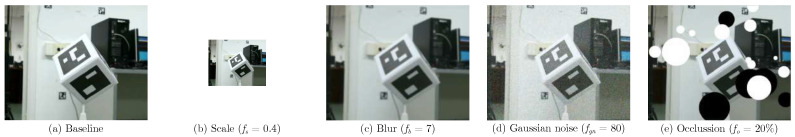
Examples of images employed in the different experiments, where the baseline images are transformed in scale, added noise, and occlusion to test the performance of the different fiducial objects.

This section presents the experiments conducted to evaluate the proposed method. Our main goal is to analyze the properties of different fiducial objects for camera pose estimation under several image conditions, such as noise, scale, and occlusion. For that purpose, we have created seven fiducial objects using polyhedric structures: a tetrahedron, cube, octahedron, hexagonal prism, dodecahedron, rhombic dodecahedron, and icosahedron (see Figure 1). They have been chosen to test a wide range of shapes with different numbers of faces. Each object was sized to fit inside a cube of volume ≈ 114 cm^3^, and we have designed custom markers that fit its faces perfectly. We have created a dataset where the objects are observed from different viewpoints. The dataset has been artificially augmented by applying different transforms (see Figure 4), namely occlusion, blur, Gaussian noise, and occlusion. Our goal is to assess the performance of each object under all these conditions. The dataset is publicly available (https://www.uco.es/investiga/grupos/ava/portfolio/fiducial-object/) (accessed on 3 December 2023) for other researchers to use it.

This section is organized as follows. In Section 4.1, we describe the experimental setup used in our study, including the equipment, materials, and procedures employed. Section 4.2 explains the baseline results using images without any added noise or occlusion. In Section 4.3, we present the results of the experiments testing different scales to evaluate the impact of the object size on the performance. Section 4.4 reports the results testing different blur levels, while Section 4.5 evaluates the robustness of the fiducial objects to different Gaussian noise levels. Then, in Section 4.6, we test the effect of occlusion. Section 4.7 compares our best fiducial object with ArUco markers. Finally, Section 4.8 summarizes all the results obtained in our experiments.

### 4.1. Experimental Setup

The seven fiducial objects of Figure 1 have been created using the method explained in Figure 3. The objects were 3D printed, and custom fiducial markers were created for each one, adapting to the available surface area and the number of bits needed to match the number of faces. Several pictures of each object were taken to obtain the three-dimensional position of its corners using the method explained in Section 3.4.

**Figure 5:**
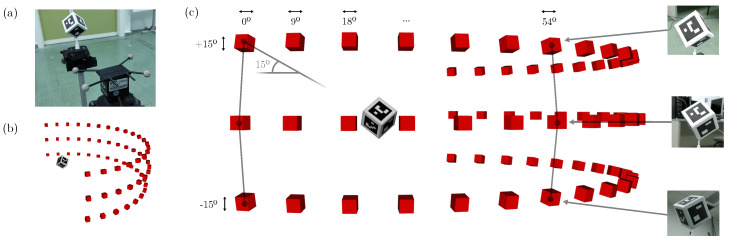
Configuration of the experiments: (**a**) image showing the camera taking an image for the dataset of one of the fiducial objects; (**b**,**c**) three-dimensional representation showing the camera in the positions employed to create the dataset.

To evaluate the performance of each object, we create our dataset in the following way. For each object, we acquired 10 images of resolution 1920×1080 pixels from 21 different positions at three different heights from the object center (see Figure 5b,c). In all cases, the distance between the camera and the object was approximately 30 cm (see Figure 5). Thus, we have 63 positions per object, making it a total of 4410=10×63×7 images in our baseline dataset. The dataset is then augmented with scaled images containing noise, blur, and occlusion, having a total set of 335.160 images in our dataset.

The camera and the object were mounted on a tripod to ensure consistent positioning across all experiments. We used a small hole in one of the corners of each object to ensure a secure fit, and this allowed us to use every face for a fair comparison. In addition, we employed a controlled environment with consistent lighting conditions to minimize sources of variability in the data collection process. Finally, we used an OptiTrack system [31] to estimate the camera and marker positions accurately and used it as ground truth.

We have not recorded the objects in movement because it is not possible for us to record video sequences where all objects undergo exactly the same trajectory. This would require a special robotic arm, which we do not have. However, the challenges associated with the movement are simulated in our tests by applying blur to the images at different levels.

A computer equipped with an AMD Ryzen 7 5800u processor with Radeon graphics and running the Ubuntu 20.04.5 OS has been employed for the experiments. On average, the computing time required to process a 1920×1080 image was 16 ms.

To estimate the performance of each fiducial object, we compared the positions estimated by our method with those of the ground truth obtained by the OptiTrack system. We employed the Horn algorithm [32] to align both reference systems. Three measures have been considered in our proposal, namely, the Average Translation Error $\bar{E_{t}}$, the Average Angular Error $\bar{E_{r}}$, and the true positive rate (TPR), which indicates the percentage of times that the object is detected in the images of the experiment.

We aim to obtain a single value (score) indicating how well an object performs compared to the others, and we achieve this by averaging individual ranking values in each one of the categories analyzed. Let us define $r_{i}^{\mu }\in \left\{1,\ldots ,7\right\}$ as the ranking value of the object *i* on the measure $\mu \in \{\bar{E_{t}},\bar{E_{r}},TPR\}$. The value $r_{i}^{\mu }=7$ indicates that object *i* obtains the best results for the measure μ, while the value $r_{i}^{\mu }=1$ indicates that it is the worst one. Then, we can define our score measure as follows:

(14) $$Score_{i}=\frac{1}{3}\sum_{\mu \in \{\bar{E_{t}},\bar{E_{r}},TPR\}}r_{i}^{\mu },$$

Note that the score values $Score_{i}$ range from 1 to 7, where 1 denotes the worst performance and 7 is the best. Please note that the scores reflect relative positions rather than absolute magnitudes of performance. Consequently, objects with identical results in terms of $\bar{E_{t}}$, $\bar{E_{r}}$ and TPR hold the same position. In such instances, the scores are averaged among them. For instance, if four objects tie in performance and rank last, they would each receive a score of 2.5, which is the average of their original scores {1,2,3,4}.

### 4.2. Baseline Experiment

**Figure 6:**
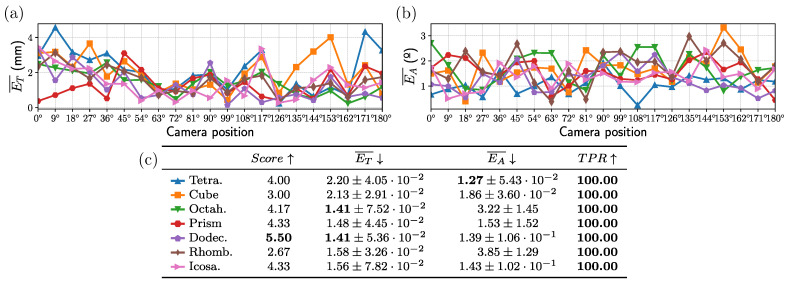
Baseline results obtained: (**a**,**b**) Median translational and angular errors in millimeters and degrees, respectively. (**c**) Table showing the performance of each fiducial object in the different measures selected for comparison. The standard deviation representing the jittering is shown next to the resulting $\bar{E_{T}}$ and $\bar{E_{A}}$. The arrow pointing upwards indicates that the higher the value, the better the result, and vice versa.

This section shows the results obtained in the images captured without applying any transform on them (i.e., no noise or scale transformation). The experiment evaluates the performance of each fiducial object under ideal conditions, with minimal sources of error or variability. The results of these experiments are presented in Figure 6. The plots in Figure 6a,b show each object’s median translational and angular errors expressed as a function of the angle between the camera and the object center. Although the errors are generally homogeneous, one can observe that the tetrahedron and the cube show higher translational errors at certain positions. The reason why is that, in these positions, a few faces of these objects are visible; thus, the number of corners to estimate the position is low, and the error increases.

Analyzing the results using these plots is difficult. This is why we employed Figure 6c to summarize the results obtained for each object in all the images captured, showing in each column its score, translational and angular errors, and the true positive rate. As can be observed, in this baseline experiment, all objects achieve low errors and there are only slight differences between them. Nevertheless, the dodecahedron seems to perform better than the rest, obtaining the highest rank, i.e., the best combination of the three measurements.

### 4.3. Scale Analysis

This experiment evaluates how the performance of each fiducial object degrades as the area of its projection in the image decreases. The projected area of an object can decrease due to (i) the object becoming smaller, (ii) the camera moving away from the object, (iii) using a camera with a lower resolution, or (iv) using a lens with a wider angle. In all these cases, the net effect is that the object becomes smaller in the image. Therefore, the simplest method to simulate these conditions is to reduce the image size by a scale factor $f_{s}$ and repeat the detections and calculations of the measurements. The results are presented in Table 1, showing the results obtained by the different objects for a given scale factor $f_{s}$ in each row. Figure 4b shows an example of a scaled image. Since the results must be compared to the baseline results (Figure 6c), they have been included as the first row of this table to ease the comparison. We also have added to Table 1 an extra row ($\bar{Score}$) with the average score in all tests. It allows us to compare the results of the different objects by summarizing their results. This value will also be shown in the following experiments since it helps to determine the best object across all the experiments.

As can be observed, the performance of the different fiducial objects does not degrade significantly until $f_{s}<0.6$. At $f_{s}=0.4$, the icosahedron is almost undetectable in any of the dataset’s images. At this level, the dodecaedron still is the best-ranked one, as well as the prism. However, as the scale is reduced further, the cube can only obtain the maximum TPR. For values of $f_{s}$ lower than 0.2, the cube’s performance also degrades and obtains low TPR values. The main conclusion that we can extract from the results is that, in general, the dodecaedron is the best one up to a certain scale point. Notably, the prism demonstrated a remarkable performance, consistently ranking either first or second in all conducted experiments and achieving the highest average rank. However, the cube is a better approach if the object needs to be observed from large distances (low observed area) since the cube markers are larger at equal volume than the ones of the dodecaedron.

### 4.4. Blurring Analysis

This section analyzes the performance of the different fiducial objects in images affected by blur. To do so, we repeated the experiments by applying different levels of blurring to the dataset images using blur box filters of increasing sizes $f_{b}$. Figure 4c shows an example of a blurred image.

The results are presented in Table 2, where the first row represents the baseline results. We can observe that the performance of the different objects decreased proportionally to the level of blurring applied, with a noticeable deterioration in the results at higher levels. Notably, the hexagonal prism, dodecahedron, and rhombic dodecahedron show particularly good results. The hexagonal prism exhibits a higher true positive rate, while the rhombic dodecahedron secured the top position under the highest blurring factor. Remarkably, the dodecahedron consistently ranked first or second across all cases examined.

### 4.5. Gaussian Noise Analysis

This section evaluates the performance of the fiducial objects to Gaussian noise. To that end, we tested the objects’ performance under various noise levels $f_{gn}$. Figure 4d shows one of the images employed in the test.

The results of this study are presented in Table 3, where the first row represents the baseline results. As can be observed, objects with more markers (located on the right side of the table) outperformed those with fewer markers. This is likely due to their increased capacity to estimate object poses as more points are used. Notably, the dodecahedrons and icosahedrons demonstrated the best performance, with the icosahedron achieving a higher true positive rate and the dodecahedron consistently ranking higher across all experiments. It is important to note that the type of noise considered in this study is random and affects different parts of the images unevenly. Consequently, objects with more faces tend to perform better, as they are more likely to detect some markers. This observation becomes more relevant as the image’s noise level increases.

### 4.6. Occlusion Analysis

This section presents the results of applying artificial occlusion to the original images. We draw artificial black and white circles on the dataset images to simulate occlusions of various degrees $f_{o}$. Figure 4e shows an example of an occluded object. The degree of occlusion $f_{o}$ is measured as the percentage of the occluded object. To ensure the validity of our results, the same circles are drawn for each object at each degree. We have created fifteen different occluded versions of the original image for each degree of occlusion $f_{o}$ to account for randomness.

The results are shown in Table 4, where the first row represents the baseline results. One can observe that the fiducial objects with more markers outperform those with fewer markers. Moreover, the dodecahedron and icosahedron performed best in these experiments.

### 4.7. Influence of the Marker Shape on the Dodecahedron’s Performance

As shown in the previous experiments, the dodecaedron is, in general, the best-performing object. In the following experiment, we compare our dodecahedron (using pentagonal markers) with a dodecaedron using ArUco (squared) markers. It is noted for reference that the pentagonal markers contain 20 bits, while the ArUco markers have 16 bits. Our goal is to analyze whether the use of a more adapted fiducial marker has any impact on performance.

**Figure 7:**
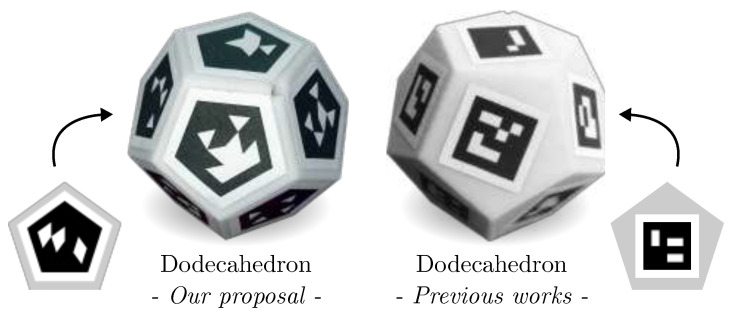
Fiducial objects developed with a dodecahedron shape and the use of pentagonal and Aruco markers.

The fiducial objects used in this experiment are shown in Figure 7. A new dataset for the two objects has been created using the same methodology as in the previous dataset, but in this case, we have reduced the number of viewpoints to 33 per object. This dataset then has a total of 330 images per object.

As in the previous case, we have evaluated the robustness of each object to changes in scale, blur, Gaussian noise, and occlusion. The results are shown in Table 5. The results for the dodecahedron with pentagonal markers differ from those in Table 1, Table 2, Table 3 and Table 4 due to the use of a new dataset for this experiment. In this case, the maximum possible value for the score is 2, since we are ranking only two objects. As can be observed, our fiducial object outperforms the previous approach in all the tests. In most cases, the precision using pentagonal markers (our solution) doubles the precision obtained using squared markers. As already indicated, our approach is able to cover better the space of the object faces so that it can be detected at larger distances. Also, since the pentagon has more corners than the square, it obtains better accuracy when estimating the pose of the object.

### 4.8. Overall Analysis

**Figure 8:**
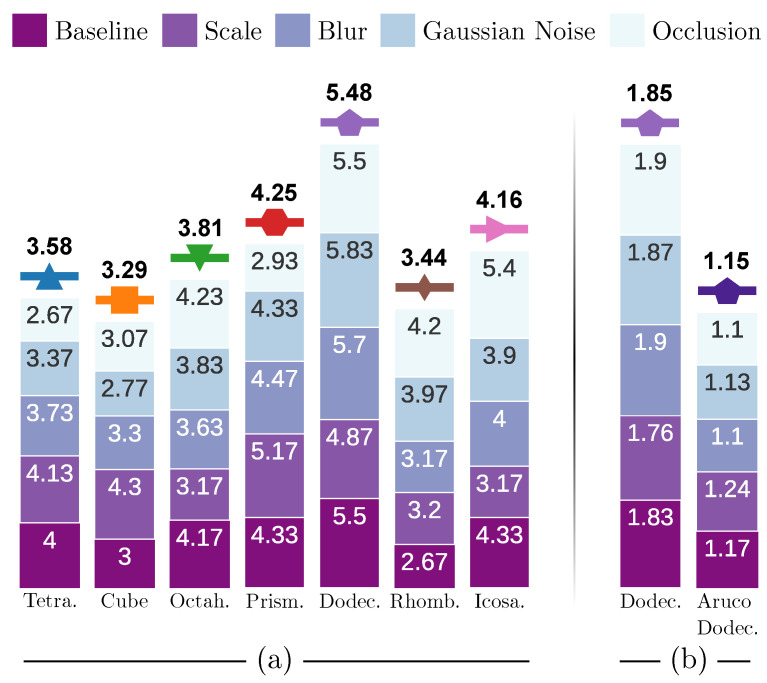
Overall analysis chart. The icon associated with each fiducial object is shown at the top of its bar. (**a**) Summary of the scores obtained for each object in the experiments conducted in Section 4.3, Section 4.4, Section 4.5 and Section 4.6. (**b**) Summary of scores obtained in experiments in Section 4.7.

Having completed the analysis of different fiducial objects under various challenging conditions, this section aims to summarize the results obtained. To do so, we have presented them in Figure 8, where, for each object analyzed, we have represented as a vertical bar the $\bar{Score}$ obtained in each experiment. At the top of the bar, we indicate the average value of these scores. As can be observed in Figure 8a, for the first set of experiments (Section 4.3, Section 4.4, Section 4.5 and Section 4.6), the dodecaedron obtains the best results, obtaining an average $\bar{Score}$ of 5.8. For the second set of experiments (Section 4.7), Figure 8b shows that, on average, our dodecahedron obtains an $\bar{Score}$ of 1.85. In conclusion, our experiments suggest that employing a dodecahedron in combination with pentagonal fiducial markers yields a superior performance compared to the other objects tested.

## 5. Conclusions

This paper has proposed a method to create custom fiducial objects for camera/object pose estimation. Our method not only enables accurate camera pose estimation but also demonstrates superior efficiency in utilizing available planar space. Furthermore, it has exhibited robustness against a variety of challenges commonly associated with such systems. Our preliminary experiments on different fiducial object configurations have provided valuable insights into the optimal polyhedron structure. Both our solution and the experimental dataset are publicly available for use by other researchers.

As for future work, several areas of potential improvement and expansion can be identified. First, this work has not focused on tracking. The system’s performance could be enhanced through the application of marker tracking algorithms between frames, which would bring additional robustness against motion blur. Second, the integration of artificial intelligence, specifically machine learning-powered state-of-the-art marker detection systems, could further enhance the detection rate of the object, particularly in challenging situations where highly accurate pose estimation is essential. Finally, the development of a method to enable the use of different fiducial objects for a single pose estimation could broaden the range of potential applications.

## Figures and Tables

**Table 1:** Results of experiments conducted with different scaling levels $f_{s}$, evaluating the method’s performance under varying distances between the camera and the fiducial objects. $\bar{Score}$ values are obtained using the results given by the factors $f_{s}\in \left[1,0.8,0.6,0.4,0.2\right]$.

Scale Level	Metric	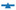 Tetra.	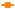 Cube	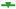 Octah.	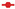 Prism	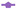 Dodec.	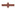 Rhomb.	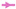 Icosa.
$f_{s}=1$	Score↑	4.00	3.00	4.17	4.33	5.50	2.67	4.33
	$\bar{E_{T}}↓$	2.20	2.13	1.41	1.48	1.41	1.58	1.56
	$\bar{E_{A}}↓$	1.27	1.86	3.22	1.53	1.39	3.85	1.43
	TPR↑	100.00	100.00	100.00	100.00	100.00	100.00	100.00
$f_{s}=0.8$	Score↑	4.00	3.00	4.17	4.33	5.17	2.67	4.67
	$\bar{E_{T}}↓$	2.24	2.10	1.41	1.46	1.41	1.61	1.56
	$\bar{E_{A}}↓$	1.31	1.86	3.47	1.71	1.56	3.91	1.42
	TPR↑	100.00	100.00	100.00	100.00	100.00	100.00	100.00
$f_{s}=0.6$	Score↑	3.33	3.33	3.00	5.67	5.67	3.67	3.33
	$\bar{E_{T}}↓$	2.22	2.13	1.82	1.59	1.50	1.61	1.87
	$\bar{E_{A}}↓$	1.71	1.87	8.05	1.61	1.65	4.26	2.42
	TPR↑	100.00	100.00	100.00	100.00	100.00	100.00	100.00
$f_{s}=0.4$	Score↑	4.33	5.17	2.00	5.50	5.50	4.50	1.00
	$\bar{E_{T}}↓$	3.24	2.57	4.09	2.11	1.75	2.12	6.36
	$\bar{E_{A}}↓$	1.91	1.92	18.06	2.01	2.23	4.31	99.21
	TPR↑	96.83	100.00	93.65	100.00	100.00	100.00	1.59
$f_{s}=0.2$	Score↑	5.00	7.00	2.50	6.00	2.50	2.50	2.50
	$\bar{E_{T}}↓$	7.77	4.42	–	4.58	–	–	–
	$\bar{E_{A}}↓$	71.83	3.46	–	9.31	–	–	–
	TPR↑	17.46	100.00	0.00	93.65	0.00	0.00	0.00
$\bar{Score}↑$		4.13	4.30	3.17	5.17	4.87	3.20	3.17

**Table 2:** Results of experiments conducted with different blurring levels $f_{b}$. $\bar{Score}$ values are obtained using the results given by the factors $f_{b}\in \left[0,1,3,5,7\right]$.

Blur Level	Metric	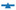 Tetra.	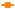 Cube	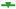 Octah.	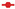 Prism	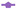 Dodec.	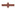 Rhomb.	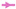 Icosa.
$f_{b}=0$	Score↑	4.00	3.00	4.17	4.33	5.50	2.67	4.33
	$\bar{E_{T}}↓$	2.20	2.13	1.41	1.48	1.41	1.58	1.56
	$\bar{E_{A}}↓$	1.27	1.86	3.22	1.53	1.39	3.85	1.43
	TPR↑	100.00	100.00	100.00	100.00	100.00	100.00	100.00
$f_{b}=1$	Score↑	2.67	3.33	4.17	4.67	5.83	2.67	4.67
	$\bar{E_{T}}↓$	2.20	2.13	1.40	1.47	1.40	1.58	1.57
	$\bar{E_{A}}↓$	2.13	1.86	3.30	1.54	1.38	3.84	1.48
	TPR↑	100.00	100.00	100.00	100.00	100.00	100.00	100.00
$f_{b}=3$	Score↑	3.33	3.00	4.33	4.33	5.67	3.00	4.33
	$\bar{E_{T}}↓$	2.19	2.08	1.37	1.50	1.41	1.57	1.63
	$\bar{E_{A}}↓$	1.67	1.85	3.35	1.75	1.40	3.83	1.47
	TPR↑	100.00	100.00	100.00	100.00	100.00	100.00	100.00
$f_{b}=5$	Score↑	4.33	2.00	3.00	3.33	6.00	4.00	5.33
	$\bar{E_{T}}↓$	2.35	2.22	1.86	1.75	1.63	1.74	1.72
	$\bar{E_{A}}↓$	1.53	1.83	8.33	1.73	1.56	3.99	1.58
	TPR↑	100.00	96.83	100.00	98.41	100.00	100.00	100.00
$f_{b}=7$	Score↑	4.33	5.17	2.50	5.67	5.50	3.50	1.33
	$\bar{E_{T}}↓$	4.43	3.39	5.32	3.12	3.56	6.11	5.91
	$\bar{E_{A}}↓$	2.60	1.87	13.74	2.00	2.10	10.91	30.32
	TPR↑	95.24	90.48	90.48	92.06	100.00	100.00	73.02
$\bar{Score}↑$		3.73	3.30	3.63	4.47	5.70	3.17	4.00

**Table 3:** Results of experiments conducted with different Gaussian noise levels $f_{gn}$. $\bar{Score}$ values are obtained using the results given by the factors $f_{gn}\in \left[0,20,40,60,80,100\right]$.

Gaussian Noise Level	Metric	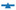 Tetra.	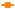 Cube	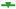 Octah.	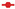 Prism	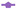 Dodec.	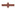 Rhomb.	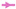 Icosa.
$f_{gn}=0$	Score↑	4.00	3.00	4.17	4.33	5.50	2.67	4.33
	$\bar{E_{T}}↓$	2.20	2.13	1.41	1.48	1.41	1.58	1.56
	$\bar{E_{A}}↓$	1.27	1.86	3.22	1.53	1.39	3.85	1.43
	TPR↑	100.00	100.00	100.00	100.00	100.00	100.00	100.00
$f_{gn}=20$	Score↑	3.33	2.17	4.33	3.83	6.33	3.00	5.00
	$\bar{E_{T}}↓$	2.20	2.15	1.49	1.50	1.42	1.62	1.59
	$\bar{E_{A}}↓$	1.60	1.84	3.50	1.58	1.38	3.87	1.44
	TPR↑	100.00	98.41	100.00	98.41	100.00	100.00	100.00
$f_{gn}=40$	Score↑	3.17	2.00	3.83	5.17	5.17	4.67	4.00
	$\bar{E_{T}}↓$	2.20	2.21	1.95	1.61	1.98	1.62	1.62
	$\bar{E_{A}}↓$	1.63	1.77	5.48	1.59	1.57	3.92	30.03
	TPR↑	96.83	93.65	98.41	96.83	98.41	98.41	98.41
$f_{gn}=60$	Score↑	3.00	3.33	3.33	4.00	6.33	4.67	3.33
	$\bar{E_{T}}↓$	2.63	2.29	1.83	1.99	1.54	1.89	2.11
	$\bar{E_{A}}↓$	1.71	1.77	4.70	2.00	1.72	4.63	5.87
	TPR↑	63.49	79.37	77.78	90.48	93.65	93.65	93.65
$f_{gn}=80$	Score↑	3.33	3.33	3.50	4.33	5.83	4.83	2.83
	$\bar{E_{T}}↓$	2.92	2.46	2.09	2.09	1.87	2.37	3.09
	$\bar{E_{A}}↓$	1.17	3.63	9.11	8.82	3.51	8.51	57.41
	TPR↑	38.10	50.79	63.49	69.84	69.84	74.60	74.60
$\bar{Score}↑$		3.37	2.77	3.83	4.33	5.83	3.97	3.90

**Table 4:** Results of experiments conducted with different occlusion levels $f_{o}$. $\bar{Score}$ values are obtained using the results given by the factors $f_{o}\in \left[0,10,20,40,60\right]$.

Occlusion Level	Metric	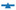 Tetra.	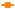 Cube	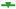 Octah.	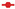 Prism	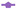 Dodec.	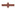 Rhomb.	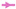 Icosa.
$f_{o}=0$	Score↑	4.00	3.00	4.17	4.33	5.50	2.67	4.33
	$\bar{E_{T}}↓$	2.20	2.13	1.41	1.48	1.41	1.58	1.56
	$\bar{E_{A}}↓$	1.27	1.86	3.22	1.53	1.39	3.85	1.43
	TPR↑	100.00	100.00	100.00	100.00	100.00	100.00	100.00
$f_{o}=10$	Score↑	1.33	3.33	3.33	3.67	6.67	4.67	5.00
	$\bar{E_{T}}↓$	2.56	2.35	1.83	2.34	1.59	1.94	2.43
	$\bar{E_{A}}↓$	10.25	4.18	12.33	6.59	1.79	4.40	2.34
	TPR↑	63.28	74.07	86.88	86.98	97.14	96.83	97.99
$f_{o}=20$	Score↑	1.33	1.67	6.00	3.33	4.33	5.33	6.00
	$\bar{E_{T}}↓$	3.50	5.13	1.90	2.36	3.02	2.01	2.24
	$\bar{E_{A}}↓$	20.47	20.37	14.84	11.91	7.32	5.28	4.26
	TPR↑	47.39	51.01	78.10	74.39	92.59	92.17	93.76
$f_{o}=40$	Score↑	2.00	3.67	3.33	1.67	6.67	4.67	6.00
	$\bar{E_{T}}↓$	3.38	2.90	3.22	3.75	2.24	3.27	2.33
	$\bar{E_{A}}↓$	32.09	24.23	37.09	40.08	9.54	9.74	10.03
	TPR↑	19.31	23.32	48.04	38.41	72.70	72.38	79.58
$f_{o}=60$	Score↑	4.67	3.67	4.33	1.67	4.33	3.67	5.67
	$\bar{E_{T}}↓$	3.50	3.88	3.13	5.61	4.83	16.62	4.24
	$\bar{E_{A}}↓$	24.01	36.57	48.62	54.90	33.20	31.05	30.78
	TPR↑	10.58	13.23	26.19	11.90	37.56	33.94	43.22
$\bar{Score}↑$		2.67	3.07	4.23	2.93	5.5	4.20	5.4

**Table 5:** Results of the experiments comparing our dodecahedron with pentagonal markers with a dodecahedron using ArUco markers (previous works). Please note that the score values range from 1 up to 2 because we are comparing only two methods.

Distorsion	Level	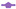 Dodec.				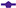 Aruco Dodec.			
		Score↑	$E_{t}↓$	$E_{a}↓$	TPR↑	Score↑	$E_{t}↓$	$E_{a}↓$	TPR↑
Baseline	1	1.83	1.17	1.20	100.00	1.17	3.27	1.95	100.00
Scales	$f_{s}=0.8$	1.83	1.25	1.55	100.00	1.17	4.18	4.50	100.00
	$f_{s}=0.6$	1.83	1.46	1.84	100.00	1.17	3.65	8.18	100.00
	$f_{s}=0.4$	1.83	1.79	3.02	100.00	1.17	5.22	10.66	100.00
	$f_{s}=0.2$	1.50	–	–	0.00	1.50	–	–	0.00
Scales $\bar{Score}↑$		1.76				1.24			
Blur	$f_{b}=1$	1.83	1.17	1.58	100.00	1.17	3.68	2.14	100.00
	$f_{b}=3$	1.83	1.22	1.39	100.00	1.17	7.56	4.73	100.00
	$f_{b}=5$	2.00	1.56	1.67	100.00	1.00	8.88	43.58	90.91
	$f_{b}=7$	2.00	4.43	1.64	100.00	1.00	9.72	14.17	81.82
Blur $\bar{Score}↑$		1.90				1.10			
Gaussian noise	$f_{gn}=20$	1.83	1.16	1.44	100.00	1.17	3.08	4.09	100.00
	$f_{gn}=40$	2.00	1.23	1.34	100.00	1.00	2.32	5.75	30.30
	$f_{gn}=60$	1.67	1.41	1.40	100.00	1.33	0.11	21.04	3.03
	$f_{gn}=80$	2.00	1.56	1.73	87.88	1.00	–	–	0.00
Gaussian noise $\bar{Score}↑$		1.87				1.13			
Occlusion	$f_{o}=10$	2.00	1.53	4.46	92.93	1.00	4.38	7.23	92.32
	$f_{o}=20$	2.00	1.69	4.66	92.53	1.00	5.50	7.25	89.90
	$f_{o}=40$	2.00	4.61	14.75	77.98	1.00	7.24	16.25	73.54
	$f_{o}=60$	1.67	4.51	22.75	75.56	1.33	7.48	14.87	74.34
Occlusion $\bar{Score}↑$		1.90				1.10			

## Data Availability

Publicly archived datasets can be found at https://www.uco.es/investiga/grupos/ava/portfolio/fiducial-object/.
